# Defining the short-term disease recurrence after loop electrosurgical excision procedure (LEEP)

**DOI:** 10.1186/s12905-020-00901-1

**Published:** 2020-02-26

**Authors:** Nicholas Papalia, Amanda Rohla, Selphee Tang, Jill Nation, Gregg Nelson

**Affiliations:** 1 Department of Obstetrics and Gynaecology, University of Calgary, 1108-1500 7th Street SW, Calgary, AB T2R 1A7 Canada; 2 Department of Obstetrics and Gynaecology, Alberta Health Services, Calgary, Canada

**Keywords:** LEEP, Follow-up, Colposcopy, HSIL, CIN2–3, HPV

## Abstract

**Background:**

The goal of cervical cancer screening is to identify dysplastic lesions for subsequent excision in order to prevent invasive disease. There is clinical equipoise, on how to best follow women for disease surveillance after treatment with some Canadian provinces exclusively performing colposcopy and some utilizing Human Papilloma Virus (HPV) testing in addition to cervical cytology. Loop Electrosurgical Excision Procedure (LEEP) is used to treat pre-invasive HPV-mediated disease and patients are typically followed for 12 months after disease excision. This study aims to quantify the prevalence of high-grade disease at the time of the second follow-up colposcopy visit, in a practice setting that utilizes laser ablation in addition to LEEP.

**Methods:**

In a retrospective cohort study, consecutive patient charts were accessed through the electronic medical record system, ARIA, at the Tom Baker Cancer Centre, in Calgary, Alberta, from January 2010 to December 2015. Data was extracted and a REDCap database was used to compile pertinent information from charts meeting inclusion criteria. Descriptive and analytic statistics were performed.

**Results:**

Of the 303 patients identified, 221 patients met inclusion criteria. 86% of these patients met discharge criteria from colposcopy after the second follow up visit. 31 (14%) were seen in a subsequent visit for abnormal findings. Of these, 7 (3.2%) underwent further treatment for high-grade disease/Cervical Intraepithelial Neoplasia (CIN 2/3). Of the 31, 23 (10.6%) had a third – negative – visit, resulting in discharge from colposcopy. One patient had a repeat LEEP for persistent Low-Grade Squamous Intraepithelial Lesion (LSIL).

**Conclusion:**

In summary, our data demonstrates a prevalence of 3.2% of high-grade disease at the time of a second colposcopic follow up visit after treatment, in a setting which frequently utilizes laser ablation in combination with LEEP, for large lesions. This recurrence rate is consistent with most published literature on recurrence rates of CIN2/3.

## Background

In Canada, cervical cancer represents 1.5% of new cancer diagnoses in women [1]. There is decreasing incidence of this disease which is likely attributable to thorough optimized screening programs and Human Papilloma Virus (HPV) vaccinations. However, approximately 400 women die annually from cervical cancer in Canada [1]. The Society of Obstetricians and Gynecologists of Canada (SOGC) 2012 guideline on managing abnormal cervical cytology recommends standard management for cervical dysplasia [2]. Women diagnosed with High Grade Squamous Intraepithelial Lesion/Cervical Intraepithelial Neoplasia (HSIL/CIN2–3) who are treated with a Loop Electrosurgical Excision Procedure (LEEP) to prevent invasive disease [3] are currently followed in colposcopy for two visits over 12 months, post-treatment, to assess for completeness of excision. The rate of disease recurrence is approximately 3–4% [4, 5].

There is limited evidence on the ideal follow-up protocol for women after they have had a LEEP. The current practice — and recommendation — in Canada is two negative colposcopy visits, with women undergoing cytology and endocervical curettage, with biopsy if necessary, at 6-month intervals. Alternatively, a negative HPV test with negative cytology at 6-months is considered adequate follow up [2]. This is based on level II-2 evidence. Currently, a randomized controlled trial is taking place in Canada, assessing the sensitivity of HPV testing at the first post procedure visit versus the traditional two-visit follow up [5].

In Calgary, HPV testing is not routinely performed in follow-up, and local recurrence rates are not available. It is not uncommon for small, central LEEPs to be combined with peripheral laser ablation of residual dysplastic tissue, theoretically minimizing the volume of cervix excised while preventing disease recurrence. There is a paucity of data on combination treatments. This study aims to quantify the prevalence of disease recurrence (HSIL/CIN 2–3) in the second visit at 12 months, after one negative follow-up visit in this unique demographic and treatment modality.

## Methods

This was a retrospective cohort study. Electronic medical records were accessed from the Tom Baker Cancer Centre’s (TBCC) electronic information system, ARIA (ARIA MO Manager v.13.7. Varian Medical Systems, Inc. Palo Alto, USA). REDCap [6] was used for data collection and tracking. Data was extracted from colposcopy forms, pathology and operative reports by NP and AR. January 2010 served as the initial LEEP data collection point. Sequential charts were accessed and assessed for inclusion criteria. A target of 292 patient charts was established based on a sample size calculation with a frequency of 5% and a 2.5% variance at the 95% confidence level. The last LEEP included in the study occurred in December 2015 in order to allow for sufficient follow up and chart completion.

LEEP procedures in Calgary are done in the outpatient setting with operating room facilities available for patients requiring sedation and anesthetic beyond a paracervical block. Immediately prior to LEEP, colposcopy is performed to identify the transformation zone with Lugol’s solution and a LEEP electrode is selected to best fit the targeted tissue to be excised. Laser is used to ablate any residual or peripheral dysplastic appearing tissue, especially with a broad (Type I) transformation zone. An endocervical curettage (ECC) is subsequently performed. In follow up visits, targeted biopsies and ECC are used when clinically appropriate without used of anesthetic.

Inclusion criteria were as follows: patients over the age of 18 with biopsy or endocervical curettage (ECC) proven HSIL/CIN2/3 with LEEP performed between January 2010 and December 2015 at the Tom Baker Cancer Centre in Calgary, Alberta. Patients were excluded if they had prior treatment for cervical cancer or dysplasia, had a concurrent diagnosis such as adenocarcinoma in-situ (AIS), or if records were incomplete due to missing data or loss to follow-up. A CONSORT diagram (Diagram 1) was completed, and STROBE cohort criteria were followed.

Once data collection was complete, the data was analyzed with SAS version 9.3 (SAS Institute Inc., Cary, NC, USA). Descriptive statistics were used to summarize baseline characteristics and follow up details. Frequencies and proportions, along with 95% confidence intervals were calculated for the rates of disease present at the second follow up visit, additional visits, and further LEEPs required. Ethics approval was obtained in 2016 by The Health Research Ethics Board of Alberta Cancer Committee (HREBA.CC-16-0429).

## Results

303 patients were included for data collection. Patients were excluded for the following reasons: inadequate follow up (67), lack of HSIL/CIN2–3 on biopsy or ECC (8), prior treatment (5), concurrent diagnosis of AIS (2). 221 patients were subsequently analyzed.

Baseline characteristics can be found in Table 1. The median age was 32 with a range from 19 to 68. Forty-one percent of the patients were nulligravid, and 53% were nulliparous. Twenty-four percent of patients identified as smokers. All patients had either a positive cervical biopsy (for CIN 2/3), a positive ECC, or both. The majority of cases did not receive concurrent laser treatment at the time of LEEP (66%) and the majority of patients had pathologic margins free of disease (54%).

**Table 1:** Baseline Characteristics

Characteristic	Frequency (%) or median (IQR)
Age in years, median (IQR)	32 (11) Range 19 to 68
Gravidity	
0	90 (40.7%)
1	40 (18.1%)
2+	91 (41.2%)
Parity	
0	117 (52.9%)
1	41 (18.6%)
2+	63 (28.5%)
Smoker	
Yes	54 (24.4%)
No	167 (75.6%)
Pre-LEEP biopsy	
Negative	10 (4.5%)
LSIL	9 (4.1%)
HSIL/SIL unqualified^a^	155 (70.1%)
Not done	47 (21.3%)
Pre-LEEP ECC	
Negative	64 (29.0%)
LSIL	4 (1.8%)
HSIL/SIL unqualified^a^	138 (62.4%)
Not done	15 (6.8%)
Laser Use	
Yes	76 (34.4%)
No	145 (65.6%)
Margins	
Margins free	120 (54.3%)
Endocervical margins	43 (19.5%)
Ectocervical margins	35 (15.8%)
Both	23 (10.4%)
ECC at LEEP	
Negative	178 (80.5%)
LSIL	3 (1.4%)
HSIL	23 (10.4%)
Inconclusive	13 (5.9%)
Not done	4 (1.8%)

^a^SIL unqualified includes ‘cannot rule out HSIL’

First follow-up data can be found in Table 2. These patients were all seen between four and eight months post-LEEP. There was no CIN 2 or 3 detected at the first follow up visit which may be a reflection of the low rate of biopsy at first follow-up visit. Biopsy was done for dysplastic appearing tissue and ECC was performed if the transformation zone was unable to be visualized. Cytology was positive for HSIL in one patient (0.5%) but was limited to low grade dysplasia (LSIL/CIN 1) on biopsy and required no retreatment to date.

**Table 2:** First (6-month) follow-up data

Characteristic	Frequency (%)
First follow-up impression	
Negative	181 (81.9%)
LSIL	11 (5.0%)
HSIL	3 (1.4%)
Not documented	26 (11.8%)
First follow-up cytology	
Negative	171 (77.4%)
ASCUS/LSIL	13 (5.9%)
ASC-H/HSIL	1 (0.5%)
Indeterminate	36 (16.3%)
First follow-up biopsy	
Negative	16 (7.2%)
CIN I or >	3 (1.4%)
Not done	202 (91.4%)
First follow-up ECC	
Negative	174 (78.7%)
LSIL or >	7 (3.2%)
Not done	40 (18.1%)

Second follow-up data can be found in Table 3. At the second follow up, 178 (80.5%) patients had negative cytology. Ninety-three percent of patients did not require biopsy at the second follow up visit. Seventy-two percent of patients had a negative ECC at this time. Fifteen percent of patients did not undergo an ECC, leaving 5% who had LSIL, and almost 3% who had HSIL. The remaining 6% were not classified due to inadequate tissue sampling.

**Table 3:** Second (12-month) follow-up data

Characteristic	Frequency (%)
Second follow-up cytology	
Negative	178 (80.5%)
ASCUS/LSIL	15 (6.8%)
ASC-H/HSIL	6 (2.7%)
Indeterminate	22 (9.9%)
Second follow-up biopsy	
Negative	8 (3.6%)
CIN 1/LSIL	5 (2.3%)
CIN2–3/HSIL	2 (0.9%)
Not done	206 (93.2%)
Second follow-up ECC	
Negative	158 (71.5%)
LSIL	11 (5.0%)
HSIL	6 (2.7%)
Indeterminate	13 (5.9%)
Not done	33 (14.9%)
Second follow-up impression	
Negative	180 (81.5%)
LSIL	11 (5.0%)
HSIL	2 (0.9%)
Not documented	28 (12.7%)
Second follow-up outcome	
Repeat colpo	28 (12.7%)
HSIL/repeat LEEP	7 (3.2%)
No disease, discharge from colpo	186 (84.2%)
Further visits	
None – Pt discharged	190 (86.0%)
Observation – no treatment to date	23 (10.4%)
Further treatment for HSIL	7 (3.2%)
Further treatment for persistent LSIL	1 (0.5%)

Based on the second colposcopy visit, the vast majority (86%) of patients were discharged from colposcopy. Seven percent of patients were diagnosed with LSIL and were seen for an additional (third) colposcopy follow-up. Six percent did not have LSIL or HSIL but were seen for an additional visit, although the current protocol would result in discharge with pathology < LSIL. 3.2% (7 patients) were diagnosed with HSIL and underwent a second LEEP.

This study found that 14.0% of patients (*n* = 31) required further follow up based on findings at the second colposcopy follow up visit. Table 4 details final outcome data. Twenty-three of these patients (10.4%) required no additional treatment during the study interval and were discharged from colposcopy. Follow up, annual cervical screening is then completed by primary care or the referring gynecologist. Seven of the 31 patients who required additional visits underwent a second LEEP for treatment of HSIL/CIN2–3, for a retreatment rate of 3.2%. One additional patient underwent a second LEEP for persistent LSIL.

**Table 4:** Treatment outcomes based on second (12-month) follow-up visit

Outcome	Frequency (%) exact 95% confidence intervals
Positive for disease at second follow-up visit^b^	7 (3.2%) 1.3 to 6.4%
Re-treatment^b^	8 (3.6%) 1.6 to 6.7%
Further visits	31 (14.0%) 9.7 to 19.3%

^a^HSIL/CIN2–3 on biopsy or ECC, out of all 221 patients

^b^Includes one patient who underwent repeat LEEP for persistent LSIL

## Discussion

Our results demonstrate a retreatment rate of 3.2% due to high grade cervical dysplasia after undergoing a primary LEEP and having no evidence of disease at first follow-up colposcopy. One tenth of patients underwent a third colposcopy assessment and did not require further treatment or colposcopy follow up. This data is a reflection of the local practice in Calgary, Alberta, Canada, where laser is used in combination with LEEP in approximately one-third (34%) of cases.

Of all patients who underwent a primary LEEP for high grade cervical dysplasia (CIN 2–3), the vast majority (86%) were discharged from colposcopic with cytologic follow-up, having two ‘negative’ post-LEEP colposcopic and cytologic assessments. Of the remaining patients, most (10.4%) were discharged from colposcopic follow up to annual cytology after the third, post-LEEP assessment. The remaining 3.2% were re-treated with a second LEEP to excise CIN 2 or 3. If all patients were discharged from colposcopy follow-up after a single negative post-LEEP assessment, necessary re-treatment would not have been achieved in a timely manner.

The rationale for use of laser ablation at the time of LEEP concerns itself with mitigating obstetrical adverse outcomes, such as pre-term labour and cervical insufficiency. By potentially limiting the volume of cervix excised, use of a smaller LEEP specimen followed by laser ablation may adequately treat superficial dysplasia while sparing cervical parenchyma. Kyrgiou et al. demonstrated a dose related response to increasing depth of tissue excision although patients with untreated CIN and pregnancies prior to excision demonstrated an increased rate of preterm birth, above the baseline population [7]. A depth of 10 mm or less should be targeted for women of reproductive age but does increase the rate of recurrence in women over the age of 35 [8, 9].

The majority of published literature on rates of treatment success and recurrence rates of CIN 2–3 indicate variable numbers to what has been documented in the Calgary Zone [10–15]. Methodology within each study was variable with regard to type of treatment, number of visits, follow-up intervals, colposcopic assessment versus cytology only, use of laser in addition to LEEP, as well as patient demographics, including HPV immunization protocols. Rates have been documented from 1.4 to 26.3% [16, 17]. This large discrepancy can be attributed to the heterogeneous methodology in the literature.

Santesso et al., in a meta-analysis of randomized control trials assessing outcomes between different modalities, documented a recurrence rate of CIN 2/3 of 5.3% on review of over 8000 patients [18]. These included cold-knife conization, LEEP, and cryotherapy but none of the literature assessed LEEP with the addition of laser ablation. An elevated rate of pre-term labor was associated with all modalities, but most notably with cold knife conization suggesting that a smaller tissue specimen may be advantageous for patients who may conceive a pregnancy.

The rate of CIN 2–3 at 12-months (3.2%) may be lower than other studies due to the combination of LEEP and laser use if a wider treatment field is targeted, the lack of HPV testing in follow up (false negative cytology, ECC, or biopsy), as HPV testing has been shown to have higher sensitivity and specificity compared to cytology alone [19]. Our study also excluded patients who had CIN2–3 documented on biopsy within six months of primary LEEP. Had these cases been included, the re-treatment rate would have been higher, similar to Wu, Ju, and Cecchini’s publications [20–22].

The rate of dysplasia that we detected may be elevated relative to Prendville’s study due to their smaller sample size (102 vs. 221 in our study), consistent with a skewed population sample [23]. Papoutsis followed patients with cytology only which also may have been responsible for producing an artificially low rate of high-grade dysplasia [9].

There are several notable strengths and limitations of our study. The health information system, ARIA, and the use of REDCap data collection software allowed for thorough record keeping and referencing. Although there were limited patient numbers, the collected and complete data facilitated accurate analysis. Another strength of our study is the minimal operator variation between colposcopists. The LEEPs included in this Calgary based study were performed by five Gynecologic Oncologists and follow-up was completed by all five members of the group. This enhances the internal validity and, in turn, may hinder the external validity as protocols vary by center.

The use of laser in combination with the LEEP may affect disease rates for multiple reasons. First, tissue destruction with a laser may treat lesions not excised, or where margins are positive. This may result in a higher treatment success than if laser was not used in combination with the LEEP. Second, the availability and use of laser may result in a smaller specimen, and in turn, positive margin status. This was mitigated by excluding patients who had evidence of disease present at the first, six-month follow-up, but may still affect the results of our study. Disease recurrence beyond 12 months with LEEP and laser ablation remains to be documented.

A power calculation resulted in a target of 292 patients. After exclusions, our sample size reached 221 data points. A larger data set would have increased the reliability and precision of our estimate of the retreatment rate. This concern was balanced with the option of including LEEPs performed in the Women’s Health Clinic in Calgary^1^; however, variations in providers, charting, and data access made this option less feasible and would have subjected our data set to more variability.

A final limiting factor for this study is the lack of documentation of HPV vaccination status or HPV genotyping, which is not currently part of the standard of care in Canada. HPV vaccinations were first offered to girls in grade 5 (aged 10/11) in Alberta in 2008. Correlating HPV vaccination status and genotyping with disease and recurrence rates may provide useful prognostic information and will be an informative relationship to investigate.

Patients should be made aware of the importance of follow up after undergoing a LEEP and counselled appropriately. Until a protocol with better outcomes is established, this standard of care can be used to predict HSIL/CIN2–3 disease rates at one year after treatment, and more accurately, in a setting which utilizes laser ablation with approximately one third of LEEPs. Future research may assess the cost effectiveness of a second visit, knowing approximately 10% of patients have a third assessment which does not result in additional dysplasia detection. Future research may also seek to assess the long-term rates of disease recurrence when LEEP is combined with user of laser vaporization.

## Conclusion

In conclusion, in women undergoing a primary LEEP for CIN 2–3, dysplasia requiring further treatment is observed in 3.2% of the population at 12-months post-treatment, after having one negative, 6-month colposcopic assessment. This finding supports the use of a two-negative colposcopy follow-up protocol for this patient population, especially when HPV testing is not widely available and remains to be proven to be superior.

## Data Availability

The datasets used and/or analysed during the current study available from the corresponding author on reasonable request.

## Abbreviations

CIN2/3: Cervical Intraepithelial Neoplasia Grade 2/3
ECC: Endocervical Curettage
HPV: Human Papilloma Virus
HREBA.CC: Health Research Ethics Board of Alberta Cancer Committee
HSIL: High-Grade Squamous Intraepithelial Lesion
LSIL: Low-Grade Squamous Intraepithelial Lesion
SOGC: Society of Obstetricians and Gynecologists of Canada
STROBE: Strength of the Reporting of Observational Studies in Epidemiology

## Footnotes

1 Laser is not used in the out-patient setting in the Women’s Health Clinic. The exclusion of patients from this setting may result in data skewness.
